# The Outcome of Critically Ill COVID-19 Patients Is Linked to Thromboinflammation Dominated by the Kallikrein/Kinin System

**DOI:** 10.3389/fimmu.2021.627579

**Published:** 2021-02-22

**Authors:** Miklós Lipcsey, Barbro Persson, Oskar Eriksson, Anna M. Blom, Karin Fromell, Michael Hultström, Markus Huber-Lang, Kristina N. Ekdahl, Robert Frithiof, Bo Nilsson

**Affiliations:** ^1^ Department of Surgical Sciences, Anesthesiology and Intensive Care, Uppsala University, Uppsala, Sweden; ^2^ Hedenstierna Laboratory, Anesthesiology and Intensive Care, Department of Surgical Sciences, Uppsala University, Uppsala, Sweden; ^3^ Department of Immunology Genetics and Pathology, Uppsala University, Uppsala, Sweden; ^4^ Department of Translational Medicine, Lund University, Malmö, Sweden; ^5^ Unit for Integrative Physiology, Department of Medical Cell Biology, Uppsala University, Uppsala, Sweden; ^6^ Institute for Clinical and Experimental Trauma-Immunology, University Hospital of Ulm, Ulm, Germany; ^7^ Linnaeus Centre for Biomaterials Chemistry, Linnaeus University, Kalmar, Sweden

**Keywords:** thromboinflammation, kallikrein/kinin system, complement system, coagulation system, fibrinolysis system, COVID-19, prognosis

## Abstract

An important manifestation of severe COVID-19 is the ARDS-like lung injury that is associated with vascular endothelialitis, thrombosis, and angiogenesis. The intravascular innate immune system (IIIS), including the complement, contact, coagulation, and fibrinolysis systems, which is crucial for recognizing and eliminating microorganisms and debris in the body, is likely to be involved in the pathogenesis of COVID-19 ARDS. Biomarkers for IIIS activation were studied in the first 66 patients with COVID-19 admitted to the ICU in Uppsala University Hospital, both cross-sectionally on day 1 and in 19 patients longitudinally for up to a month, in a prospective study. IIIS analyses were compared with biochemical parameters and clinical outcome and survival. Blood cascade systems activation leading to an overreactive conjunct thromboinflammation was demonstrated, reflected in consumption of individual cascade system components, e.g., FXII, prekallikrein, and high molecular weight kininogen and in increased levels of activation products, e.g., C4d, C3a, C3d,g, sC5b-9, TAT, and D-dimer. Strong associations were found between the blood cascade systems and organ damage, illness severity scores, and survival. We show that critically ill COVID-19 patients display a conjunct activation of the IIIS that is linked to organ damage of the lung, heart, kidneys, and death. We present evidence that the complement and in particular the kallikrein/kinin system is strongly activated and that both systems are prognostic markers of the outcome of the patients suggesting their role in driving the inflammation. Already licensed kallikrein/kinin inhibitors are potential drugs for treatment of critically ill patients with COVID-19.

## Introduction

The COVID-19 pandemic caused by SARS-CoV-2, a coronavirus first reported at the end of 2019 in Wuhan, China, has had a tremendous socioeconomic impact on human society across the globe. In most cases SARS-CoV-2 causes an influenza-like disease of mild-to-medium severity, but early on it became clear that in some cases, an increased risk of deadly thromboembolism developed that required high doses of low-molecular (LMW) heparin (150-300IU/kg) to prevent thrombosis (1). In a few cases the disease worsened after 9-10 days and developed into a full-blown acute respiratory distress syndrome (ARDS)-like pulmonary inflammation with endothelialitis, thrombosis, and vascular angiogenesis that has often required intensive care with ventilator support (2) and that is characterized by a general cyto-/chemokine dysregulation (3–5), increased risk of thromboembolism, reduced oxygen saturation, and signs of severe pulmonary damage revealed by chest computed tomography, such as ground glass opacities (6, 7).

In the blood, the innate immune system consists of several cascade systems that include the complement, contact, coagulation, and fibrinolytis systems, together with cellular defense systems such as leukocytes [including polymorphonuclear cells (PMNs), monocytes and NK cells], platelets, and endothelial cells. Here we will refer to all these components together as the intravascular innate immune system (IIIS) (8, 9). The IIIS acts as a purging system that identifies and removes microorganisms and activated apoptotic and necrotic cells and debris. It also orchestrates the subsequent immune/thromboinflammatory responses that lead to the repair of injured tissue (10). In some cases the IIIS overreacts giving rise to a severe thromboinflammation that causes collateral tissue damage. Examples of such thrombotic reactions are those that occur in cardiac infarction or stroke, during hemodialysis and in the setting of transplantation during ischemia-reperfusion injury and vascular rejection (11, 12). However, the cause of the COVID-19 -linked lung injury is not yet defined, but indirect evidence speaks in favor of a thromboinflammatory reaction triggered by the virus itself and more likely by infected and damaged cells (see several recent review articles) (1, 13–21).

Negatively charged surfaces such as the biological lipid membranes of necrotic and apoptotic cells as well as viruses can activate both the complement system *via* the classical (C1q) and lectin [mannose binding lectin (MBL), ficolins, collectins] pathways (the CPW and LPW, respectively) as well as *via* the contact (FXII) system (22–25). Pre-pandemic and recent studies of CoV-1 and -2 have indicated that these viruses can trigger complement activation *via* their spike proteins and the virus-infected host cells (26–28). The SARS CoV-1 virus has already been reported to cause an ARDS-like condition and in animal models complement C3-knockout (KO) mice infected with SARS CoV-1 recover more easily and have a much milder disease course than do wild-type mice (29). Also, increased plasma levels of sC5b-9 complexes have been found in COVID-19 patients, deposition of C4d and C3 has been detected in the lungs of diseased patients and binding of MBL and MBL associated serine protease (MASP)-2 of the LPW to virus proteins of the SARS CoV-2 virus has been reported (26, 30, 31).

Generation of the anaphylatoxins C3a and C5a by the complement system and bradykinin (BK) production by the kallikrein/kinin system are involved in ARDS of bacterial sepsis origin and may be drivers of the COVID-19 ARDS-like lung injury (32, 33). All of these peptides have chemotactic properties, induce increased vascular permeability, and activate immune and endothelial cells leading to inflammation and cyto-/chemokine expression. In ARDS, the anaphylatoxins and BK mediate leukocyte infiltration, particularly of PMNs, and mediate the leakage of fluid into the intercellular spaces between alveolar epithelial cells and the endothelium leading to reduced gas exchange between the alveolar space and the blood. A similar mechanism may be involved in SARS-CoV-2-induced ARDS. COVID-19 patients have an increased risk of both venous and arterial thrombosis. Activation of neutrophilic granulocytes by the anaphylatoxins that cause neutrophil extracellular trap (NET) formation, exposes both tissue factor (TF) and FXII in COVID-19 and may be one mechanism by which thrombi are formed (34, 35). We also recently reported that high levels of the recognition molecule MBL is strongly associated with thrombotic disease in COVID-19 patients, perhaps by recognizing damaged cells in this milieu (36).

An important property of the IIIS is that it discriminates self from non-self and is therefore a major trigger of inflammation. The aim of our investigation was to determine to what degree the IIIS is activated in COVID-19 in a naïve population with ARDS-like disease in the beginning of the pandemic. Our goal is to pinpoint the mechanisms underlying the lung damage associated with COVID-19, identify new biomarkers and targets for therapeutic intervention, and obtain support for giving already-licensed therapeutic inhibitors of these systems (licensed for angioedema) to patients with COVID-19 ARDS. The results of our comprehensive investigation of the IIIS-associated cascade systems in COVID-19 patients support the concept that all these cascade systems (particularly the kallikrein/kinin system) and cellular responses that constitute the IIIS are engaged in a full-blown thromboinflammatory reaction that damages organ systems in COVID-19 patients, not only the lungs but also other organs such as the kidneys and heart.

## Material and Methods

### Patients

A prospective single-center observational study was performed in the ICU of a mixed surgical and medical unit at Uppsala University Hospital, a tertiary hospital in Uppsala, Sweden. The first 70 patients >18 years of age with confirmed or suspected COVID-19 admitted to the ICU between March 13 and April 30, 2020 were screened for inclusion. COVID-19 was diagnosed by positive reverse-transcription polymerase chain reaction (RT-PCR) on nasopharyngeal swabs. Of the screened patients, 66 were included on the basis of a positive PCR for SARS-CoV-2, informed consent, and the availability of a blood sample taken at admission to the ICU. Nineteen of the first included patients were followed for up to one month, the only exclusion criterion being extracorporeal circulation therapies since such treatments are known to induce substantial activation of the IIIS (12).

Apart from the customary ICU care and medications, all patients received thromboprophylaxis with either dalteparin sodium at 117 IU/kg [91–152 (median and interquartile range)] or apixaban at 5 mg (n = 1) to target factor Xa (target range 0.2–0.5 kIU/L) for thrombosis prophylaxis and at 200 IU/kg to target factor Xa (target range 0.5–1.0 kIU/L) for treatment after thromboembolic events. Blood was sampled in EDTA-tubes, centrifuged, and stored as plasma at −70°C until analyzed.

Clinical data were recorded prospectively, including medical history, medications, physiological data, and date of death. The Simplified Acute Physiology Score 3 (SAPS-3) (37), Sequential Organ Failure Assessment (SOFA) score (38), and renal function “Kidney disease improving global outcomes” (KDIGO) (39), circulatory support, and respiratory support data were collected as detailed in the Results section (**Table 1**).

**Table 1:** Patient demographic characteristics and comorbidities.

	All patients (n = 66)^#^
**Women, n (%)**	15 (23)
**Age, years**	60 (52–70)
**Body weight, kg**	85 (77–98)
**BMI, kg/m^2^**	28.4 (25.6–32.8)
**SAPS-3 score**	51 (47–58)
**COVID-19 day on ICU arrival**	10 (8–12)
**ACEi/ARB treatment, n (%)**	25 (38)
**Anti-coagulant treatment prior to COVID-19, n (%)**	12 (18)
**30-day mortality, n (%)**	18 (28)
**Invasive ventilation, n (%)**	41 (63)
**Renal replacement therapy during ICU stay, n (%)**	7 (11)
**Comorbidities, n (%)**	
Pulmonary disease	16 (25)
Hypertension	35 (54)
Heart failure	3 (5)
Peripheral vessel disease	10 (15)
Previous thromboembolic event	5 (8)
Diabetes mellitus	19 (29)
Malignancy	4 (6)

^#^Data are expressed as n (%) or median [interquartile range (IQR)]. SAPS-3, simplified acute physiology score 3; ICU, intensive care unit; ACEi/ARB, angiotensin converting enzyme inhibitor/angiotensin receptor blocker.

The study was approved by the Swedish National Ethical Review Agency (EPM; No. 2020-01623). Informed consent was obtained from the patient or the next of kin if the patient was unable to give consent. The Declaration of Helsinki and its subsequent revisions were followed. The protocol for the study was registered (ClinicalTrials ID: NCT04316884); STROBE guidelines were followed for reporting.

### General Clinical Chemistry Analyses

All routine lab tests were performed at the hospital’s clinical chemistry department. Blood samples were collected on admission to the ICU and daily during the ICU stay. Full blood counts (FBC), C-reactive protein (CRP), and kidney and liver function tests were performed in the hospital’s central laboratory. FBC was analyzed using a Sysmex XN™ instrument (Sysmex, Kobe, Japan); plasma CRP, ferritin, troponin I, procalcitonin, and kidney and liver markers were analyzed on an Architect ci16200 (Abbott Laboratories, Abbott Park, IL, USA). Acute kidney injury (AKI) was defined according to the KDIGO AKI definition. IL-6 was measured by commercial sandwich ELISA kit (D6050, R&D Systems, Minneapolis, MN, USA).

### Complement Analyses

Complement function of the CPW and alternative pathway (APW) were measured by hemolytic assays (40). The concentrations of C3, C4, and factor B were quantified by nephelometry, MBL by ELISA, and C1q by a magnetic bead-based assay (41). The activation products were measured as follows: C3a by ELISA using monoclonal antibody (mAb) 4SD17.3 for capture and biotinylated polyclonal anti-C3a for detection (42), C3d,g by nephelometry after removal of high molecular weight forms of C3 by PEG precipitation (43), C4d by ELISA using an anti-human neo-C4d epitope mAb for capture (SVAR Life Science, Malmö, Sweden) (44), and sC5b-9 by an in-house ELISA using anti-human neo-C9 mAb aE11 for capture and polyclonal anti-C5 for detection (42). In order to compensate for intra-individual differences in C3 concentration due to consumption or acute phase reaction, the ratios of C3a to C3 (C3a/C3) and C3d,g to C3 (C3d,g/C3) were calculated.

### Coagulation/Kallikrein/Kinin System Analyses

Thrombin-antithrombin (TAT) complexes were analyzed by ELISA using a pair of matched antibodies: anti-human thrombin for capture and anti- human antithrombin (ATIII) for detection (TAT-EIA, Enzyme Research Laboratories, South Bend, IN, USA). D-dimer was measured with STA Liatest D-Di Plus using STA-R Max2 (Diagnostica Stago, Saint-Ouen-l’Aumône, France). The kallikrein/kinin system proteins FXII, high molecular weight kininogen (HK) and prekallikein were all measured using an automated Western blot-like assay (WES). Simple Western 12- to 230-kDa assay cartridges were used under reducing conditions with a WES^®^ analyzer (Protein Simple, Santa Clara, CA, USA) according to the manufacturer’s manual. The HK concentration represents an indirect measure of BK generation. The samples were diluted 1/250 (prekallikrein) and 1/500 (FXII, HK) in 0.1 × WES Sample Buffer, and the proteins were detected using affinity-purified, biotin-labeled goat anti-human FXII IgG (GAFXII-AP, Enzyme research Laboratories, South Bend, IN, USA) and goat anti-human kininogen IgG (BAF1396, R&D Systems, Minneapolis, MN, USA), and affinity-purified and peroxidase-conjugated anti-human prekallikrein IgG (SAPK-APHRP, Enzyme Research Laboratories). The electrophoretic protein separation and immunodetection that followed were performed using the default SimpleWestern™ settings. The quantified immune-detected signal, i.e., the area under the curve was analyzed using Compass software (version 4.0.0. ProteinSimple™), which also was used to convert the electropherograms into virtual blots. For each assay, a pool of EDTA-plasma from five healthy donors with assigned values for FXII (30 mg/L), HK (70 mg/L) and prekallikrein (50 mg/L) (45) was serially diluted and used as standard curve to calculate individual values in the patient and control samples.

Kallikrein-C1inhibitor (KK-C1INH) complexes were measured by a slightly modified sandwich ELISA as described before (46) using an affinity purified polyclonal sheep anti-human prekallikrein antibody (SAPK-AP, Enzyme research Laboratories) for capture and a biotinylated polyclonal rabbit anti-human C1INH antibody (in-house) followed by streptavidin-HRP (GE Healthcare) for detection. Standards were made from *in vitro* generated complexes of KK-C1INH (using a molar excess of C1INH) diluted in freshly drawn lepirudin anticoagulated plasma.

For all assays, EDTA-plasma from healthy blood donors was used as controls. The controls were handled and stored as the patients’ samples.

### Statistics

GraphPad Prism 8.4 (GraphPad software, San Diego, CA, USA), Statistica (ver 13.5, TIBCO Software Inc., Palo Alto, CA, USA), or R (version 4.0.2) were used for statistical analyses and to generate graphs. Given the number of observations and their non-normal distribution, non-parametric statistical tests were used for all analyses. The Mann-Whitney U test was used to calculate differences between groups. Correlations according to Spearman were used to assess dependence between variables. Chi-square analysis was used to evaluate the risk of RAS inhibitors. Fisher´s exact test was used to evaluate patients’ results in relation to a given reference interval representing the normal range based on mean ± 2SD (as calculated in the clinic). Kaplan-Meier plots were used to estimate the probability of survival.

To investigate if and to what extent respiratory and renal failure mediate the activation of IIIS an uni- and a multivariable logistic regression was performed. Organ failure or organ support were dependent variables with IIIS activation and age as predictors to calculate odds ratios. SAPS-3 was included as a measure of illness severity. As the number of observations were limited, we used age as a surrogate for the pre COVID-19 risk of death. Moreover, if the IIIS activation variable was a predictor of outcome in the univariate model we assessed if it was still an independent predictor after adjusting for age. A p-value of <0.05 was considered significant, * <0.05, ** <0.01, *** <0.001 and **** <0.0001.

## Results

### COVID-19 Patients Admitted to the ICU

Blood was drawn prospectively from 66 patients when admitted to the ICU; 19 of these patients were followed longitudinally for up to 1 month. Descriptive data and the general history of the patients from the first day of admission are summarized in **Table 1**.

### Evidence of an Established Thromboinflammation on Day 1

**Figure 1:**
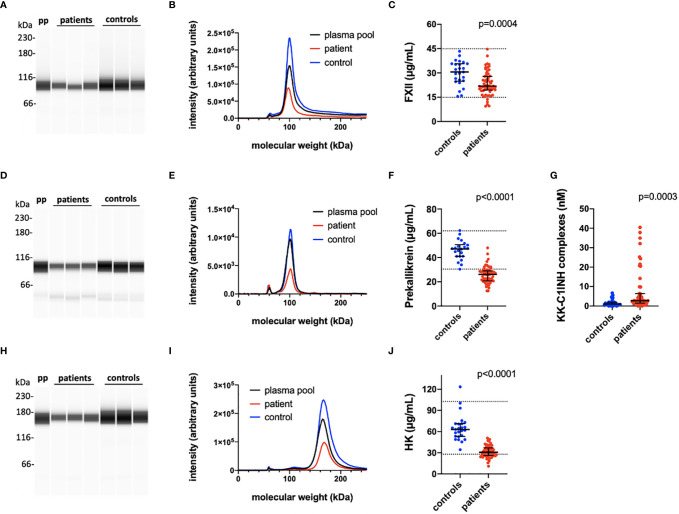
Quantification of the kallikrein/kinin proteins FXII, prekallikrein and high molecular weight kininogen (HK). The kallikrein/kinin system was activated as reflected in high consumption of FXII, prekallikrein, and HK. EDTA plasma from COVID-19 patients and controls were analyzed by capillary electrophoresis under reduced SDS-PAGE-like conditions and the specific kallikrein/kinin proteins detected by specific antibodies against FXII, prekallikrein, and HK, respectively **(A**, **D**, **H)**. The area under the curves of the scan of the chemiluminescence intensities plotted in the panels **(B, E, I)** were interpreted to their specific concentration by comparing the value for each peak with a standard curve specific for each protein **(C, F, J)**. The concentration of KK-C1INH complexes were analyzed by sandwich ELISA (panel **G**). Representative patients and controls are shown in panels **(A, B, D, E, H, I)**. The Mann-Whitney U test was used to calculate differences between the groups. For more details see *Materials and Methods*. pp, plasma pool; KK, kallikrein; C1INH, C1inhibitor; HK, high molecular weight kininogen.

**Figure 2:**
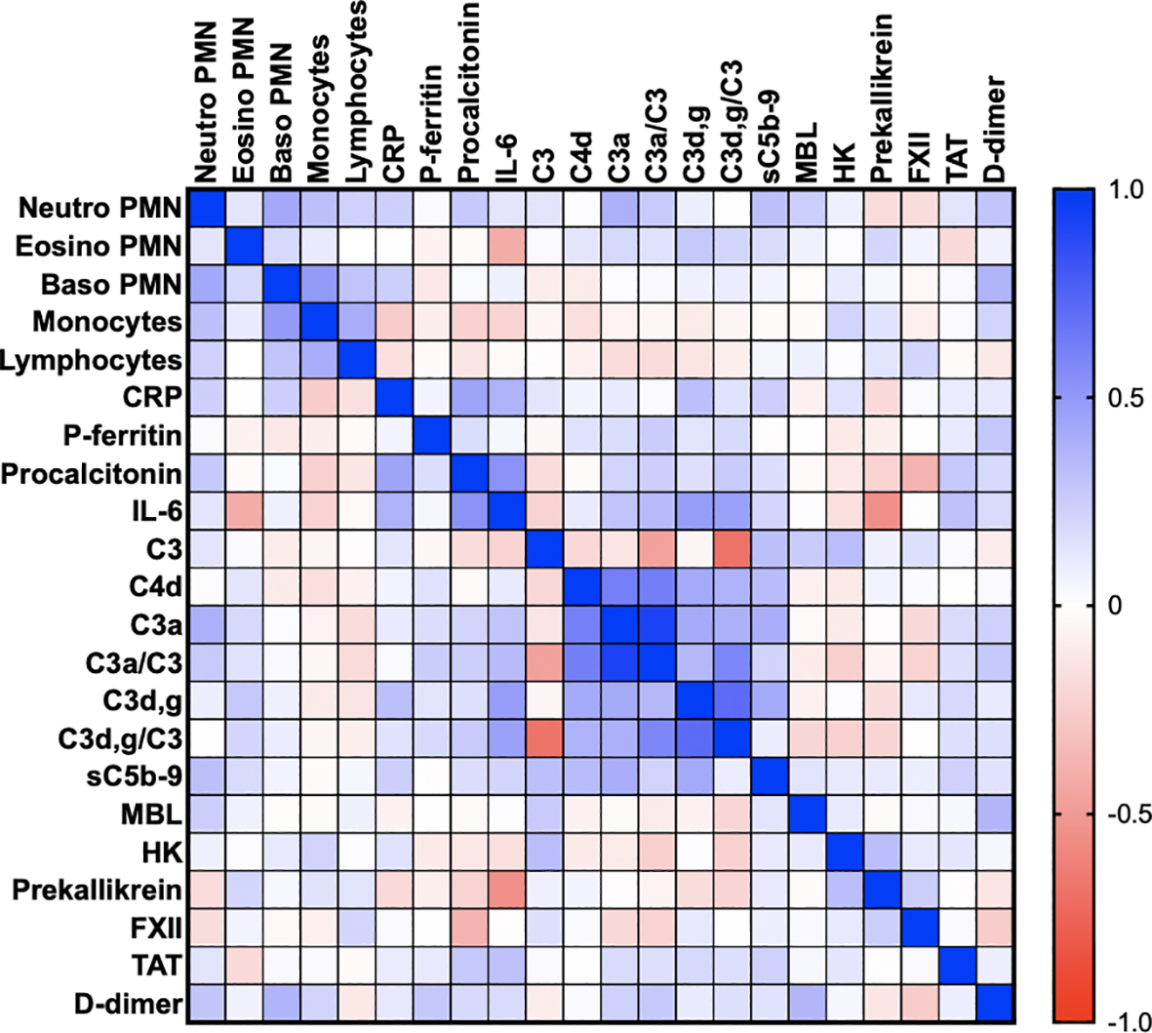
Thromboinflammation activation in critically ill COVID-19 patients. Thromboinflammatory parameters from 66 consecutive patients with COVID-19 were analyzed by the Spearman correlation test and presented in a heat map. Multiple significant correlations within each cascade system and also between the cascade systems, blood cell counts, CRP, and IL-6 were found. A summary of the most important correlations is presented in **Table 3**. TAT, thrombin-antithrombin.

**Table 2** is a summary of the results from the quantitation of the specific IIIS biomarkers on the day of admission to the ICU. Most of the markers showed values that were outside the reference ranges. These markers include all the cascade systems of the IIIS (the complement, coagulation, contact, and fibrinolysis systems) as well as ferritin, procalcitonin, CRP, and IL-6. In particular, the kallikrein/kinin system was strongly activated as reflected in high consumption of FXII, prekallikrein, and HK (**Figure 1**) (47). The consumption of prekallikrein was accompanied by generation of the corresponding complexes between the activation product KK and C1INH. Multiple correlation analyses showed that there were a number of significant correlations both within each cascade system and also between the cascade systems, immune cell counts, CRP levels, and IL-6 levels (heatmap, **Figure 2**). These correlations are further emphasized in **Supplemental Table S1**, which shows that the IL-6 and CRP levels are correlated with markers of both the complement and kallikrein/kinin systems. All these findings demonstrate that the IIIS is engaged in a response that is translated into thromboinflammation.

**Table 2:** Thromboinflammatory parameters in serum/plasma at admission to the ICU (n ≤ 65).

	n	Median		IQR	Reference range	p
**General inflammation:**						
IL-6	37	103	⇡	46–167	<7.0 ng/L	****
CRP	63	169	⇡	118–235	<5 mg/L	****
Ferritin	52	1,074	⇡	515–2,477	25 - 310 µg/L	****
Procalcitonin	59	1.05	⇡	0.19–1.10	<0.05 µg/L	****
Neutrophil count	56	5.8	⇡	3.95–7.58	1.5–5.4 × 10^9^/L	****
Platelet count	64	214	⇢	147–300	150–350 × 10^9^/L	****
**Complement system:**						
Classical pathway^1^	22	98	⇢	82–112	80%–120%	**
Alternative pathway ^1^	22	107	⇢	78–125	50%–150%	ns
C1q^2^	65	91	⇣	68 –111	70–300 mg/L	***
MBL^2^	65	625	⇡	303–1,112	288–611 kU/L	****
C4^2^	22	0.23	⇢	0.19–0.39	0.13–0.32 g/L	**
C3^2^	65	1.21	⇢	0.97–1.46	0.67–1.29 g/L	****
Factor B^2^	22	0.61	⇡	0.53–0.70	0.19–0.50 g/L	****
C4d^3^	55	1,966	⇡	551–3,815	<1,000 ng/ml	****
C3a^3^	65	253	⇡	164–390	<200 µg/L	****
C3d,g^3^	65	6.8	⇡	5.7–8.3	<5.3 mg/L	****
sC5b-9^3^	65	142	⇡	103–213	<50 µg/L	****
**Kallikrein/kinin system:**						
FXII^2^	62	21.9	⇣	19.4–27.9	14.9–44.9 mg/L	ns
Prekallikrein^2^	65	26.1	⇣	20.7–29.0	30.4–62.0 mg/L	****
HK^2^	62	30.6	⇣	26.0–36.9	27.8–102.6 mg/L	****
**Coagulation system:**						
APTT^1^	16	38	⇢	33–40	30–42 s	ns
INR^1^	20	1.1	⇢	1.0–1.2	0.9–1.2 INR	ns
Fibrinogen^2^	11	6.5	⇡	5.1–7.6	2.0–4.2 g/L	****
TAT^3^	64	47	⇡	33–67	<10 µg/L	****
**Fibrinolysis system:**						
D-dimer ^3^	61	1.40	⇡	0.88 – 2.65	<0.50 mg/L	****

^1^Functional assays of the cascade systems in plasma.

^2^Plasma protein concentration.

^3^Activation product plasma concentration.

Fisher´s exact test was used. IQR, interquartile range; MBL, mannose binding lectin; HK, high molecular weight kininogen; APTT, activated partial thromboplastin time; INR, international normalized ratio; TAT, thrombin-antithrombin.

** <0.01, **** <0.0001.

**Table 3:** Correlations between thromboinflammatory parameters within the IIIS (n ≤ 66).

		*r*	p	n
**Neutrophilic PMN**	C3a	0.388	**	54
	C3a/C3	0.266	*	54
	sC5b-9	0.314	*	54
	D-dimer	0.293	*	54
**Basophilic PMN**	D-dimer	0.369	**	52
**Procalcitonin**	FXII	−0.384	**	55
	TAT	0.276	*	57
**IL-6**	C3a/C3	0.34	*	36
	C3d,g	0.479	**	36
	C3d,g/C3	0.483	**	36
	Prekallikrein	−0.556	***	37
**CRP**	C3d,g	0.321	**	63
	sC5b-9	0.245	*	63

TAT, thrombin-antithrombin.

### Thromboinflammatory Reactions in Serial Samples

**Figure 3:**
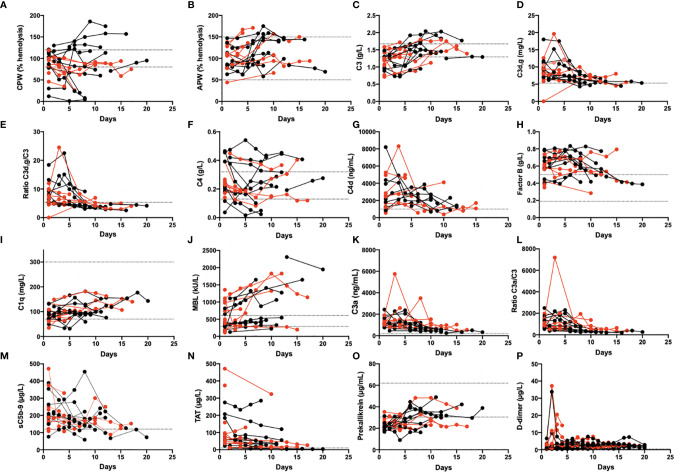
Longitudinal monitoring of thromboinflammation activation in critically ill COVID-19 patients. Analyzed complement related parameters included activation by the CPW and APW **(A, B)**. levels of C3, C3d,g and the C3d,g/C3 ratio **(C, D, E)**, C4 and C4d **(F, G)**, Factor B, C1q, and MBL **(H, I, J)**, the activation markers C3a, the C3a/C3 ratio, and the sC5b-9 complex **(K, L, M)**. Additional parameters included the levels of TAT **(N)**, prekallikrein **(O)**, and D-dimer **(P)**. Red dots: patients who died; black dots: patients who survived. The dotted lines mark the reference range. The presented data are from 19 of the first included patients that were followed for up to 1 month. CPW, classical pathway of complement; APW, alternative pathway of complement; MBL, mannose binding lectin; TAT, thrombin-antithrombin.

Several of the biomarkers were followed over time in 19 patients (**Figure 3**). At admission, some of their plasma protein levels and the functional tests showed signs of complement and kallikrein system consumption (e.g., CPW, C1q, and prekallikrein). Concomitantly, several of the activation markers were elevated including C4d, C3a, C3d,g, sC5b-9, D-dimer, and TAT; all these values tended to normalize over time in the ICU.

The parameters that precede C3 activation (C3d,g/C3 ratio) were investigated over time for possible correlations, and the results are presented in **Table 4** and **Supplemental Figure S1**. Even though a varying number of observations are included from each patient, this table indicates how the CPW function, the recognition molecules C1q for the CPW and MBL for the LPW, were correlated with the first common activation step (C4) in the cascade. This supports the conclusion that activation of both the LPW and the CPW had occurred. Interestingly, levels of prekallikrein were also found to be correlated with C3d,g/C3 and factor B levels (*r* = −0.27; p < 0.05; **Supplemental Figure S1**), suggesting an association between the kallikrein/kinin system and the APW. C1q, prekallikrein, and C3d,g/C3 levels also showed an association with TAT levels (*r* = −0.39; p < 0.001; **Supplemental Figure S1, I**), suggesting a link between complement and coagulation.

**Table 4:** Correlations between C3 activation (C3d,g/C3) and parameters of the various complement pathways in COVID- 19 patients that provided serial samples (n = 19).

	Analyses	*r*	p
**Functional tests**	CPW	−0.42	****
	APW	−0.11	****
**Plasma proteins**	C1q	−0.51	***
	MBL	−0.30	***
	C4	−0.42	****
	Prekallikrein	−0.34	**
**Activation fragment**	C4d	0.35	**

CPW, classical pathway; APW, alternative pathway; MBL, mannose binding lectin.

### Association of Complement and Kallikrein/Kinin Activation With Survival in the ICU

**Figure 4:**
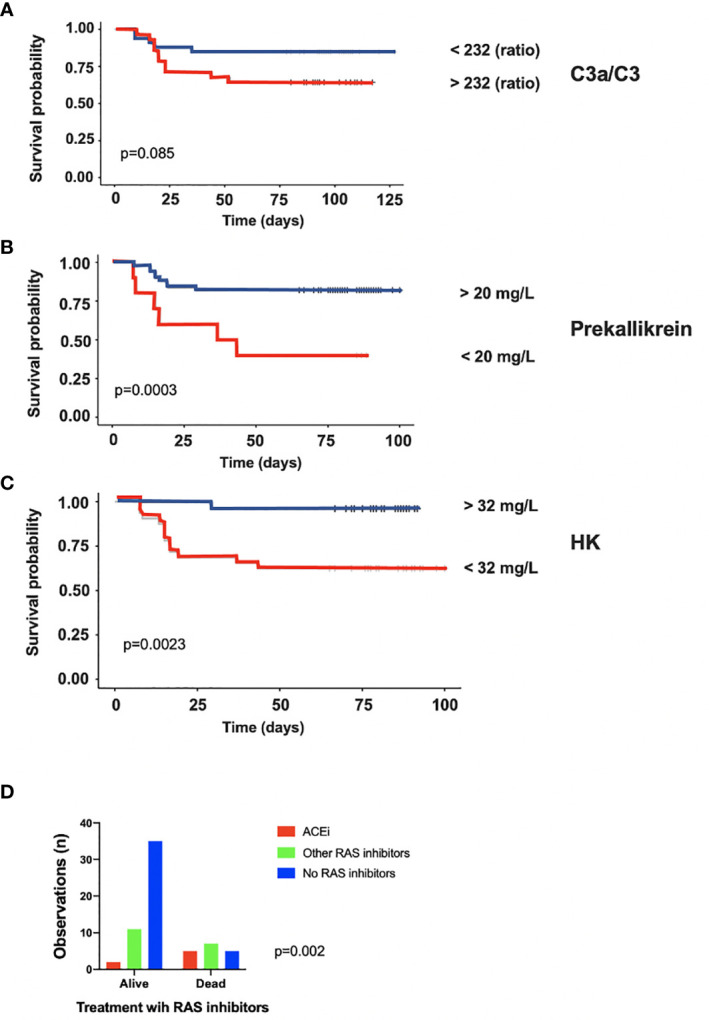
Association of intravascular innate immune system activation in COVID-19 ARDS with death. Kaplan-Meier plots of C3a/C3 **(A)**, Prekallikrein **(B)** and HK **(C)** further stressed, that both the complement and the kallikrein/kinin systems are more activated in the patients who later died during their stay in the ICU. In panel **(D)** it is shown that the proportion of patients who died during the ICU stay was much higher in the group treated with ACE-1 inhibitors than among the patients on other types of renin-angiotensin system (RAS) inhibitors and those that did not take any RAS inhibitors at all. The presented data are from 66 consecutive patients with COVID-19 and for statistical evaluation non-parametric Mann Whitney U, Chi^2^ and Kaplan-Meier tests were used. HK, high molecular weight kininogen; ACEi, ACE-1 inhibitors; RAS, renin-angiotensin system.

IIIS parameters at admittance were analyzed with regard to survival. The complement activation marker C3a (**Supplemental Figure S2A**) and the ratio C3a/C3 (not shown) were significantly higher in patients who died during their stay in the ICU (p = 0.03). Also, HK (**Supplemental Figure S2B**) and prekallikrein (not shown) levels were significantly or tended to be lower (p = 0.01 and p = 0.06, respectively) due to consumption (activation) in patients who died compared to those that survived during their stay in the ICU indicating that the kallikrein/kinin system was more activated in the patients who died. Thus, both the complement and the kallikrein/kinin system results predicted a poor outcome in the ICU. We then further analyzed the ratio C3a/C3, prekallikrein, and HK over time with regard to survival by using Kaplan-Meier plots (**Figures 4A–C**). Using the optimal cut-off values of 232 (ratio), 20 mg/L and 32mg/L for C3a, prekallikrein, and HK, respectively, we demonstrated that C3a/C3 levels tended to predict 30-day mortality, and prekallikrein and HK consumptions were strongly predictive of death in the studied cohort.

In order to evaluate the possible effect of ACE-1 inhibitors on survival in the cohort, patients on ACE-1 inhibitors (n = 7) were compared to those receiving other renin-angiotensin system (RAS) inhibitors (n = 19) or other hypertensive drugs and those not receiving RAS inhibitors (n = 40; **Figure 4D**). The risk of death was highest for those on ACE inhibitors, whereas those on other RAS inhibitors or non-RAS drugs all had a substantially lower risk (OR 8.8 (range 1.5–46); p < 0.01).

### Association of Complement and Kallikrein/Kinin System Activation With Organ Function

**Figure 5:**
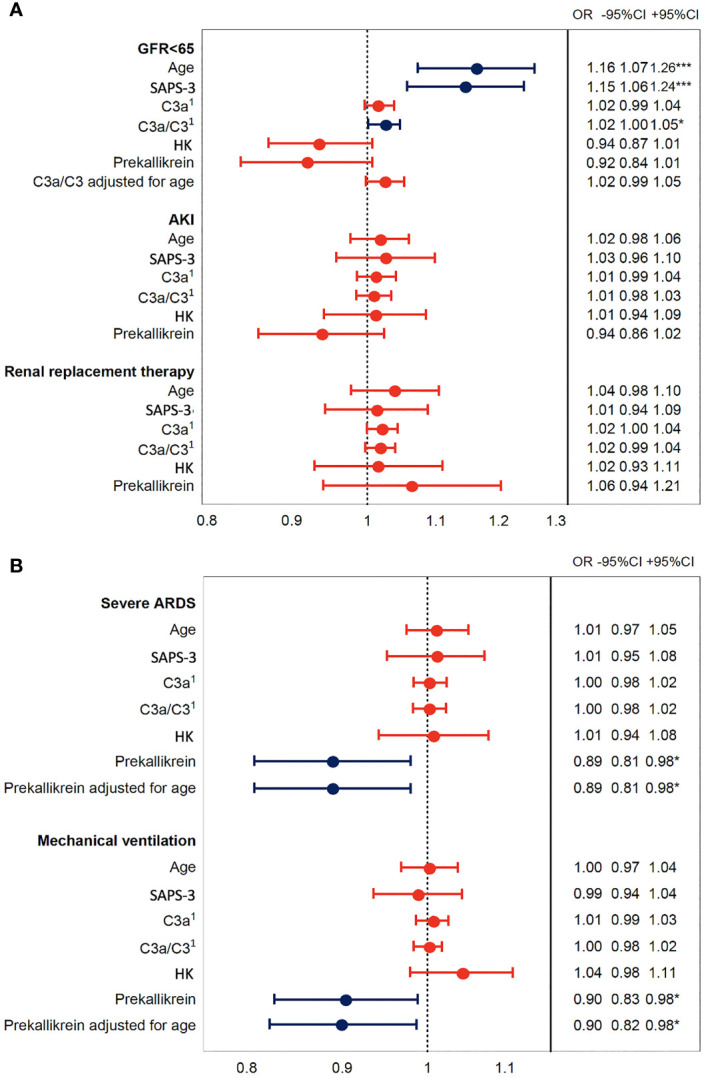
Association of IIIS activation in COVID-19 ARDS with organ damage. The plot shows odds ratios for IIIS activation variables C3a, C3a/C3, HK, and prekallikrein levels that were associated with death in **Figure 4**. Age is included as a surrogate for the pre COVID-19 risk of death and SAPS-3 as a marker of illness severity. Panel **(A)** depicts the kidney failure related outcomes, low glomerular filtration rate (GFR < 65 ml/min), acute kidney injury (AKI, vs. no AKI), and renal replacement therapy, i.e., dialysis. Panel **(B)** depicts the respiration failure related outcomes severe acute respiratory distress syndrome (ARDS, vs. mild or moderate ARDS) and mechanical ventilation. Blue intervals indicate statistical significance (*, *** right hand table), while red intervals do not. Increasing C3a/C3 ratios were predictors of low GFR but not after adjustment for age, while decreased prekallikrein levels were associated with respiratory failure also after adjustment for age. ^1^C3a has the unit of 10 ng/ml and C3a/C3 of ×10 in this figure. IIIS, intravascular innate immune system; HK, high molecular weight kininogen; SAPS-3, Simplified Acute Physiology Score 3.

Increased C3a/C3 ratio was associated with impaired renal function as assessed by estimated glomerular filtration rate [eGFR (creatinine)] (**Figure 5A**), but this effect was not seen after adjustment for age. Decreased prekallikrein level was a predictor of severe ARDS and of mechanical ventilation, and this was consistent also after adjusting for age (**Figure 5B**). These findings could suggest that the association between the C3a/C3 ratio and death is mediated through renal failure (36), and strongly suggest that the association between prekallikrein and death is mediated through respiratory failure.

### Association of Complement and Kallikrein/Kinin System Activation With ICU Disease Indices

In **Supplemental Table S2** it is shown that markers of complement and kallikrein/kinin activation correlate significantly to indices of kidney injury (AKI) and eGFR[creatinine], eGFR[cystatin C], cardiovascular disease (P-N-termpBNP, troponin I, heart rate), pulmonary function (pO_2_/FiO_2_), and with the two ICU indices SAPS-3 (probability of death) and SOFA (organ failure). D-dimer was also linked to heart rate and troponin I levels.

## Discussion

The ARDS-like COVID-19-mediated lung injury is a severe and life-threatening condition that develops in a subset of SARS-CoV-2-infected patients. The pathophysiological mechanism involved in the development of this condition is not fully elucidated, but several reports have suggested the involvement of disturbances in the cascade systems of the blood (7, 27, 30, 48–50). In the present investigation we report the results of biomarker measurements reflecting IIIS activation and thromboinflammation in a single-center study of the first eligible 66 patients admitted to our ICU at the beginning of the pandemic. The investigational patients represent essentially naïve COVID-19 patients who, in addition to receiving established ICU treatment, had only been treated with LMW heparin, minimal extracorporeal treatment (hemodialysis), and no steroids. The patients’ clinical parameters were analyzed prospectively both cross-sectionally at admission (**Table 2**) and longitudinally (**Figure 3**). The most prominent aberration in the IIIS analyses was a strong kallikrein/kinin systems activation where individual components of the kallikrein/kinin system (FXII, prekallikrein and HK) were found to be very low in patients on day 1 reflecting a very strong activation (**Figure 1**). Over time, the prekallikrein concentrations tended to normalize. The complement parameters showed mixed results, with complement function (CPW and APW hemolytic assays) and individual components ranging from high to low values, reflecting both acute-phase reactions and complement consumption (activation). Complement activation products (C3a, C3d,g, and sC5b-9) tended to be high at the beginning of the ICU treatment but normalized over time; the coagulation system activation product TAT and the fibrinolysis marker D-dimer behaved in the same way. Thus, we found a general activation of the blood cascade systems (contact, complement, coagulation, and fibrinolysis systems), indicating a complete thromboinflammatory reaction mediated by the IIIS. General cross-cascade system correlations showed multiple associations between the cascade systems. Correlations between several markers of the complement and kallikrein/kinin systems, platelets, PMNs, ferritin, IL-6, and CRP indicate that the activation of the cascade systems had been translated into inflammation. Consistent with such an important role for inflammation in COVID-19, is the finding that both IL-6 and CRP are strong markers of COVID-19 disease activity (51). Correlation of the longitudinal parameters implies that the complement activation (C3d,g/C3) was triggered by both the classical (C1q) and the lectin (MBL) pathways. Interestingly, prekallikrein consumption also correlated with APW activation (which is reflected in C3d,g/C3 generation and the factor B concentration), consistent with previous *in vitro* findings in which kallikrein was found to cleave factor B to Ba and Bb in a manner similar that of factor D (52, 53).

We found that changes in the levels of individual components and activation products of the complement and kallikrein/kinin systems were associated with survival/death, organ function, and scores of illness severity. Within the complement system, there are several strong inflammatory products, such as C3d,g and the anaphylatoxins C3a and C5a. We measured C3a and C3d,g as well as sC5b-9 as an indirect measure of C5a generation, since C5a tends to bind to its highly expressed receptors and compromise the interpretation of its measurement (54). In particular, C3a and C3a/C3 at the time of admission showed a statistically significant and strong link to death and respiratory failure, assessed by the pO_2_/FiO_2_ ratio; to eGFR(creatinine); and to heart rate (**Supplemental Figure S2** and **Table S2**). In addition, C3d,g and sC5b-9 were associated with the SAPS-3 score, with more favorable SAPS-3 levels being seen at low levels of complement activation. This finding suggests that complement activation reflects the outcome and damage to organs such as the lungs, the kidneys and the heart. A Kaplan-Meier analysis of C3a/C3 also tended to predict survival in the present cohort and logistic regression showed that C3a/C3 was associated with eGFR(creatinine) albeit not after adjustment for age.

Even more pronounced correlations and links were found for the kallikrein/kinin system. Both prekallikrein and HK were consumed (i.e., activated) to a significantly greater extent in the patients who later died during their stay in the ICU. Further supporting the predictive value of kallikrein/kinin system parameters, a Kaplan-Meier analysis demonstrated that prekallikrein and HK consumption was strongly predictive of death in the cohort. The kallikrein/kinin system was correlated with organ failure, reflected by eGFR (cystatin C), and injury reflected by troponin I levels and the propensity to receive mechanical ventilation, indicating that the lungs, kidneys and heart were affected. Logistic regression analysis of prekallikrein strongly suggested that the association with death was due to is respiratory failure. The strong correlation between FXII, prekallikrein, and HK indicated that the HK activation product BK is involved in the organ damage (34, 36, 50).

C3a, C5a and, in particular, BK formation could explain many of the symptoms and signs elicited by cell destruction, leukocyte infiltration, increased vascular permeability, thrombotic reactions and angiogenesis, which together cause poor gas exchange. This would explain the endothelialitis, thrombi, and increased vascular angiogenesis observed in pulmonary biopsies in COVID-19 patients (2). A recent *in vitro* mechanistic study of the RAS in cells obtained from bronchial alveolar lavage of COVID-19 patients strongly corroborates this concept. Due to low expression of ACE-1 and increased levels of ACE-2, the BK activity is predicted to be highly elevated (55). In turn, it is also tempting to speculate, that enhanced levels of BK and its interaction with both the BK-receptor 1 and 2 may result in disassembly and loss of intercellular adherence and tight junction molecules and consequently in an air-blood-barrier or remote organ-blood-barrier dysfunction (56, 57). Also, in this context hypertension has been suspected to be associated with an increased risk for severe COVID-19 infection, and particular drugs that act within the RAS have been suspected to increase the risk for severe disease. However, when the renin, ACE-1, and angiotensin receptor inhibitors have been grouped together in studies, this link to COVID-19 severity has not been confirmed. Given the disparate mechanisms of action of these drugs, it is necessary to compare the effects of these drugs separately. ACE-1 inhibitors lower the blood pressure by blocking ACE-1 from metabolizing angiotensin I to angiotensin II. ACE-1 also acts as an inactivator of BK, which makes it feasible that an inhibitor of ACE-1 might increase the levels of active BK and thereby aggravate the ARDS condition in COVID-19 patients. In our small cohort, we have separated our patients into those treated with ACE-1 inhibitors alone and those who are on other RAS inhibitors or on other hypertensive drugs. When we do so, we find that the risk for death substantially increases in patients on ACE-1 inhibitors, as compared to the other group without ACE-1 inhibitors. However, a larger population is required to confirm this preliminary finding.

**Figure 6:**
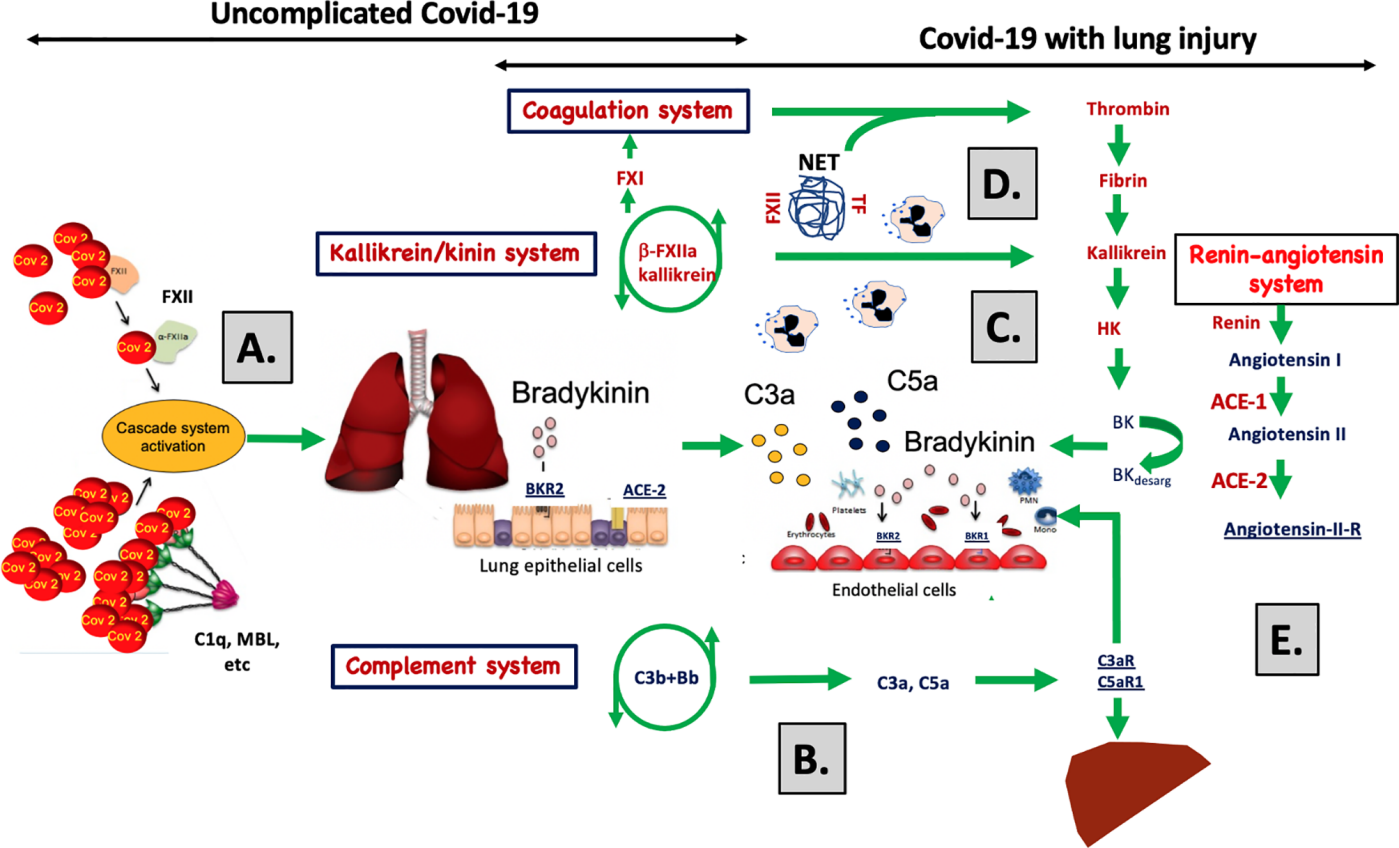
A graphical abstract summarizing the discussion of the data obtained in the study. **(A)** SARS-CoV-2 infects cells *via* ACE-2 and C1q, MBL and FXII recognize virus particles and their products, and recognize apoptotic and necrotic cells. These reactions are tentative triggers of the classical and lectin pathways of complement and of the kallikrein/kinin system described in the present study and by others (1, 13–21). The elicited C3a, C5a, and bradykinin (BK) generation induce, increased vascular permeability, endothelial activation, and nerve end stimulation *via* bradykinin receptor (BKR) 2 (15). **(B)** In response to C3a and C5a, proinflammatory cyto-/chemokines (e.g., IL-1β, IL-6) are produced that induce an acute inflammation and an acute phase reaction that increases the plasma protein (e.g., fibrinogen, MBL, C4, C3, Factor B) concentrations multi-fold and aggravate cascade system activation. **(C)** C5a triggers the up-regulation of BKR1, which is not constitutively expressed. BK can now act *via* BKR1 and BKR2 on both the pulmonary epithelium and endothelium. Together C3a, C5a, and BK have the potential to cause vascular leakage, edema, leukocyte chemotaxis, and activation that disturb oxygenation of the blood. **(D)** Local thrombi and pulmonary emboli further aggravate the oxygenation and cause collateral damage in other organs. Activated endothelial cells can induce thrombosis by binding MBL, FXII and by exposing tissue factor (TF) (34, 36, 50). Activation of neutrophilic granulocytes cause formation of neutrophil extracellular traps (NETs) that bind TF and triggers FXII activation (34, 35) and thereby initiating both the extrinsic and intrinsic pathways of the coagulation system. **(E)** The docking protein for SARS-CoV-2 on human cells is ACE-2 of the renin-angiotensin system (RAS) may cause dysregulation of the kallikrein/kinin system since BK and its metabolites are regulated directly and indirectly by the enzymes (ACE-1 and -2) of the RAS. In support of this, two of the signs and symptoms of COVID-19 can be induced as side effects during treatment of hypertension with ACE-1 inhibitors: these side effects include dry cough and increased vascular permeability (angioedema). Both of these symptoms can be alleviated by giving the patient icatibant (Firazyr^®^), an inhibitor of BKR2, suggesting that these symptoms may be caused by the kallikrein/kinin system.

Although the activation of IIIS in COVID-19 induced ARDS has many common features with ARDS of other ethiologies, such as sepsis, there are several differences both in terms of activation pathways and the focus of inflammation. We report that the IIIS is primarily activated through the kallikrein/kinin system and the CPW and LPW of complement (**Figure 6**). Others have reported the APW and later the CPW to be the key steps in complement activation in septic shock (58). These differences may be at least partly explained by the specific pathophysiologic process in SARS-CoV-2 infection, compared to the heterogeneous microbiology in different forms of sepsis. Another important dissimilarity between COVID-19 and sepsis induced ARDS is the focus of inflammation. ARDS in COVID-19 originates from the lung, as opposed to sepsis induced ARDS that is, unless the infection focus is in the lung, caused by a systemic inflammatory response in a distant organ (59). In the former, usually termed pulmonary ARDS, the lung is the motor of IIIS activation with highest IIIS activation locally, while in the latter, usually termed extrapulmonary ARDS, the lung is only secondarily affected and IIIS activation measured in plasma is less dependent on the severity of ARDS.

The concept that activation of the IIIS is either caused by the virus directly or, more likely, caused by NET formation (34, 50) in combination with the large amounts of activated and damaged (apoptotic and necrotic) cells produced, may imply that individual components of the IIIS can be targeted in order to treat COVID-19 patients. There are several licensed drugs that affect components of the IIIS. The complement system has already been targeted (31). Initial trials with a licensed anti-C5 antibody (eculizumab) have been performed, with potentially promising results (16, 60–63). Also, several anti-complement drugs are under development for use in COVID-19, such as C3 inhibitors of the compstatin family, which recently was shown to induce recovery and a drop in several major inflammatory parameters (63, 64). From the results of the present study, inhibition of the kallikrein/kinin system seems to be an important step in the search for therapeutic alternatives for treatment of COVID-19. Drugs for the treatment of angioedema target the kallikrein/kinin system: the effect of BK can be inhibited by the bradykinin receptor (BKR)-2 inhibitor icatibant (65), and the kallikrein inhibitory antibody lanadelumab (13). The kallikrein inhibitor ecallantide, which is licensed in the US, and purified and recombinant C1INH are other examples of drugs that could potentially be used in COVID-19 patients (65). The successful treatment of ARDS with icatibant in a hantavirus infection has already shown that the kallikrein/kinin system played a major role in this patient’s infection and that a BKR2 inhibitor alleviated the virus-induced ARDS (66).

A limitation in this hypothesis-generating study is the relatively low number of patients. Likewise, as our study is exploratory, we did not correct for multiple testing in the statistical analysis, which could lead to spurious associations. Importantly, the main findings of our study, e.g., kallikrein/kinin system activation and its relation to survival, were confirmed by multiple individual biomarkers, corroborating these results.

Furthermore, a clear strength is the extensive work-up leading to high resolution investigation of the IIIS. We therefore suggest that the newly described strong activation of the kallikrein/kinin system is a main driver of the ARDS-like condition in COVID-19, which is part of a conjunct activation of the IIIS. The strong link to respiratory failure and death implicates activation of the IIIS and the kallikrein/kinin system as potential mechanism that mediates the tissue damage in COVID-19 patients (Discussion summarized in **Figure 6**). The biomarkers of the IIIS, particularly within the kallikrein/kinin system, are likely to have a predictive value and pinpoint potential new targets for the treatment of COVID-19 disease with already licensed drugs.

## Data Availability Statement

The raw data supporting the conclusions of this article will be made available by the authors, without undue reservation.

## Ethics Statement

The studies involving human participants were reviewed and approved by the Swedish National Ethical Review Agency (EPM; No. 2020-01623). Informed consent was obtained from the patient or the next of kin if the patient was unable give consent. The Declaration of Helsinki and its subsequent revisions were followed. The protocol for the study was registered (ClinicalTrials ID: NCT04316884); STROBE guidelines were followed for reporting. The patients/participants provided their written informed consent to participate in this study.

## Author Contributions

ML, BP, OE, MH, MHL, KE, RF, and BN were responsible for the design of the study. ML, MH, and RF were responsible for the patients and for the collection of the patient samples. BP, OE, KE, and BN were responsible for laboratory data. BP performed the WES assays and KF performed the assessment of KK-C1INH complexes. AB provided the C4d analysis. BN wrote the first original draft of the manuscript. All authors participated in the data collection and in revision of the manuscript. ML and BP share the first authorship and RF and BN share the last authorship. ML appears first and BN appears last in the author list due to applying a strict alphabetical order for these positions in the author list. All authors contributed to the article and approved the submitted version.

## Funding

The study was funded by grants from SciLifeLab/The Knut and Alice Wallenberg Foundation (KAW2020.0182), the Swedish Research Council (2014-02569, 2014-07606, 2015-06429, 2016-01060, 2016-04519, 2020-05762), the Swedish Heart-Lung Foundation (HLF 2020-0398), and by faculty grants form Linnaeus University as well as in part by the DFG-grant CRC1149 A01 (INST 40/479-2).

## Conflict of Interest

AB is named as inventor in a patent application including claims to use of C4d as biomarker.

The remaining authors declare that the research was conducted in the absence of any commercial or financial relationships that could be construed as a potential conflict of interest

## Abbreviations

ACE, Angiotensin converting enzyme; AKI, Acute kidney injury; APTT, Activated partial thromboplastin time; APW, Alternative pathway; ARB, Angiotensin receptor blocker; ARDS, Acute respiratory distress syndrome; BK, Bradykinin; BKR, Bradykinin receptor; C1INH, C1 inhibitor; CPW, Classical pathway; eGFR, Estimated glomerular filtration rate; FBC, Full blood count; HK, High molecular weight kininogen; ICU, Intensive care unit; IIIS, Intravascular innate immune system; INR, International normalized ratio; IQR, Interquartile range; KDIGO, Kidney Disease: Improving Global Outcomes; KK, Kallikrein; KO, knock-out; LMW, Low-molecular weight; LPW, Lectin pathway; mAb, Monoclonal antibody; MASP, Mannose binding lectin Associated Serine Protease; MBL, Mannose binding lectin; NET, Neutrophil extracellular trap; PMN, Polymorphonuclear cells; RAS, Renin-angiotensin system; RT-PCR, Reverse transcription polymerase chain reaction; SAPS-3, Simplified Acute Physiology Score 3; SOFA, Sequential Organ Failure Assessment; TAT, Thrombin–antithrombin; TF, Tissue factor; WES, Western blot-like assay.
