# Supplementary material for: The Outcome of Critically Ill COVID-19 Patients Is Linked to Thromboinflammation Dominated by the Kallikrein/Kinin System
Source: Front Immunol. 2021 Feb 22;12:627579. doi: 10.3389/fimmu.2021.627579 (PMC7937878; doi:10.3389/fimmu.2021.627579)
Supplement: Supplementary file 1 [file DataSheet_1.docx]

Supplemental Fig. S1

**
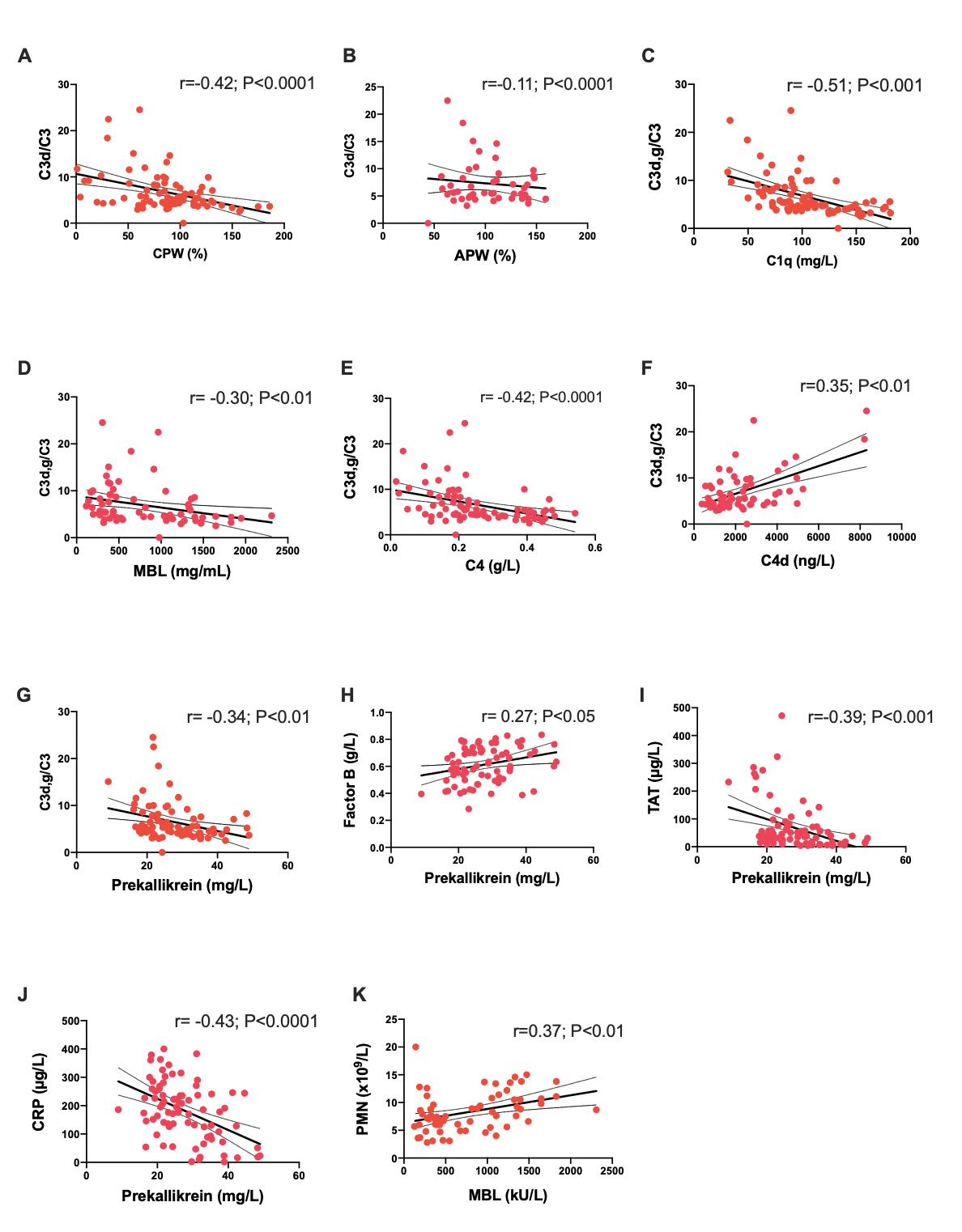
**

**Fig. S1 Correlation between thromboinflammatory parameters followed longitudinallly in critically ill COVID-19 patients.**

Correlations of thromboinflammatory parameter from 19 of the first included patients that were followed for up to a month. Spearman multiple correlation evaluation was performed. Abbreviations, CPW: classical pathway of complement; APW: alternative pathway of complement; MBL: mannose binding lectin; TAT: thrombin-antithrombin.

**
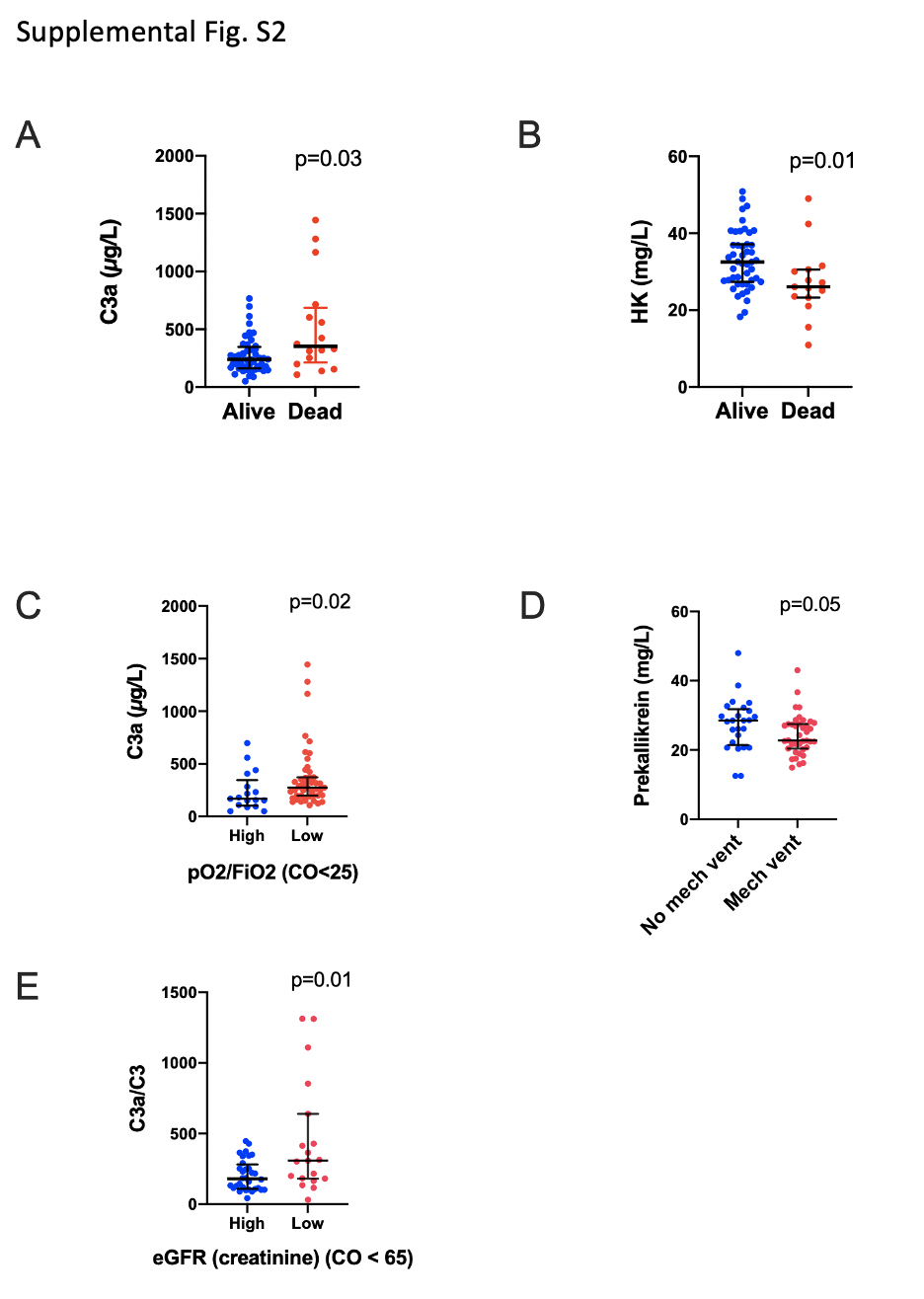
**

**Fig. S2 Association of IIIS activation in COVID-19 ARDS with death and organ failure.**

Activation of C3 monitored as C3a generation and the consumption of HK at admission was significantly higher in patients who later died compared to those that survived (A and B) (median [IQR]). C3a and C3a/C3 levels were higher when the oxygen saturation in the blood was low. as indicated by the pO_2_/FiO_2_ ratio ( C) and when the glomerular filtration rate eGFR (creatinine) was low (D). Consumption of prekallikrein was higher in the group of patients who received mechanical ventilation (E). Abbreviations, HK: high molecular weight kininogen.

**Supplemental Table S1: Correlations between various thromboinflammatory cells and components**

* p≤ 0.05; ** p≤ 0.01; *** p≤ 0.001

Abbreviations,

MBL: mannose binding lectin;

HK: high molecular weight kininogen;

TAT: thrombin-antithrombin.

**
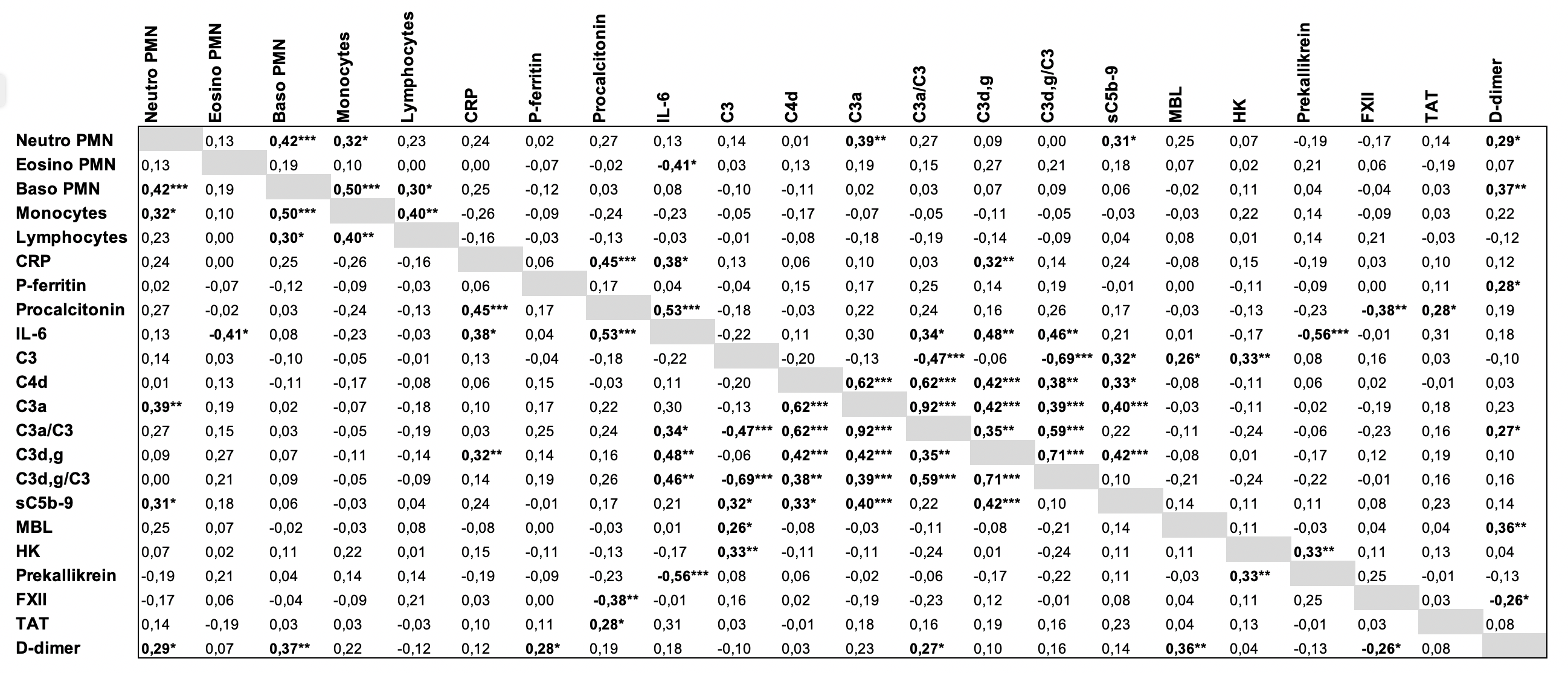
**

**Supplemental Table S2:** Correlations between thromboinflammatory parameters and organ injury (n ≤ 66)

|  |  |  |  | ***r*** | **p** | **n** |
| --- | --- | --- | --- | --- | --- | --- |
| **Acute kidney injury** | C3 | GFR[creatinine]^a^ | | 0.42 | ** | 43 |
|  |  | AKI 1^b^ |  | 0.45 | ** | 37 |
|  |  |  |  |  |  |  |
|  | PK | GFR[Cystatin C] | | 0.31 | * | 41 |
|  |  |  |  |  |  |  |
|  | HK | AKI 1^b^ |  | 0.32 | * | 36 |
|  |  |  |  |  |  |  |
| **Cardiovascular injury** | C3 | P-N-termpBNP | | -0.30 | * | 46 |
|  |  |  |  |  |  |  |
|  | C3a | Heart rate |  | 0.42 | * | 64 |
|  |  |  |  |  |  |  |
|  | FXII | Troponin I |  | -0.45 | *** | 59 |
|  |  |  |  |  |  |  |
|  | D dimer | Troponin I |  | 0.31 | * | 59 |
|  |  | Heart rate |  | 0.25 | * | 62 |
|  |  |  |  |  |  |  |
| **Pulmonary injury** | HK | pO2/FiO2 |  | 0.29 | * | 55 |
|  |  |  |  |  |  |  |
| **ICU index (death/organ failure)** | C3d,g | SAPS-3^c^ |  | -0.30 | * | 63 |
|  |  |  |  |  |  |  |
|  | sC5b-9 | SAPS-3^c^ |  | -0.29 | * | 63 |
|  |  |  |  |  |  |  |
|  | HK | SAPS-3^c^ |  | -0.25 | * | 61 |
|  |  |  |  |  |  |  |
|  | C4d | SOFA day 1^d^ |  | -0.35 | * | 55 |
|  |  |  |  |  |  |  |
|  | FXII | SOFA day 3 ^d^ |  | -0.34 | ** | 60 |
|  |  |  |  |  |  |  |
|  | PK | SOFA max ^d^ |  | -0.39 | ** | 61 |
|  |  |  |  |  |  |  |
|  |  |  |  |  |  |  |
|  |  |  |  |  |  |  |

* p≤ 0.05; ** p≤ 0.01; *** p≤ 0.001

^a^ Estimated glomerular filtration rate for creatinine or cystatin C.

^b^ The day that the AKI index 1, 2 or 3 is reached.

^c^ SAPS at arrival to ICU: ICU scoring systems for predicting mortality.

^d^ SOFA score: ICU scoring system for assessing organ damage at the ICU. Either on day 1, 3 or the maximum score during the ICU-stay.
